# Association between dietary protein intake and grip strength among adults aged 51 years and over: What We Eat in America, National Health and Nutrition Examination Survey 2011-2014

**DOI:** 10.1371/journal.pone.0191368

**Published:** 2018-01-24

**Authors:** Suruchi Mishra, Joseph D. Goldman, Nadine R. Sahyoun, Alanna J. Moshfegh

**Affiliations:** 1 Department of Nutrition and Food Science, University of Maryland, College Park, Maryland, United States of America; 2 United States Department of Agriculture, ARS, Beltsville Human Nutrition Research Center, Beltsville, Maryland, United States of America; Taipei Veterans General Hospital, TAIWAN

## Abstract

**Introduction:**

Distributing daily protein intake evenly across meals (∼25–30g/meal) has been suggested to improve muscle mass. The aim of this research is to examine the association between grip strength, total protein intake and its distribution across day’s meals in older adults.

**Methods:**

Nationally representative dietary intake data of adults aged 51 years and older (n = 4,123) who participated in What We Eat in America, NHANES 2011–2014 were analyzed. Protein intake per day and per eating occasion (breakfast, lunch, dinner, and snack) were determined. Combined grip strength was calculated and expressed in kilograms. Grip strength of individuals consuming ≥25g protein at 1 eating occasion was compared with those consuming same level of protein at 2 and 3 or more eating occasions. Grip strength of individuals in quartile 1 of daily protein intake was compared to those in the other quartiles. All associations were examined without and with adjustment for age, race/ethnicity, physical activity, health status, and smoking status. The comparison involving eating occasions and protein intake quartiles were further adjusted for daily protein intake and energy intake, respectively.

**Results:**

Only 33% of men and 19% of women had protein intake of ≥25g at 2 or more eating occasions. These individuals also had higher grip strength and daily protein intake. Grip strength was positively associated with consumption of ≥25g protein at 2 eating occasions as compared to consumption of same level of protein at 1 eating occasion (p<0.05) in unadjusted model, but not when adjusted. Grip strength was positively associated with daily protein intake among women in quartiles 3 and 4 (p<0.05) of protein intake in both unadjusted and adjusted models compared to lowest protein intake. Among men, grip strength was associated with daily protein intake in quartiles 3 and 4 (p<0.05) in the unadjusted model, but not when adjusted.

**Conclusion:**

In a nationally representative sample of older adults, consuming ≥25g protein at 2 or more eating occasions was not associated with grip strength. However, higher daily protein intake was positively associated with grip strength in women.

## Introduction

Age-related decrease in muscle mass is a major public health burden because it can cause impaired function and physical disability leading to increased morbidity and mortality [1, 2]. Adequate dietary protein intake is crucial for muscle protein synthesis to preserve muscle mass and limit the risk of disability associated with age [3, 4]. Studies have shown that higher dietary protein intake is associated with higher physical performance [5] and lower hip-fracture, potentially from improved muscle strength [6]. Although recent studies suggest beneficial effect of high protein intake on muscle strength, growing evidence suggests that a balance between protein synthesis and breakdown is not only influenced by the total protein intake but also by the pattern of protein intake. It is proposed that maintaining a threshold of protein intake (~25-30g/ meal) across the day’s intake may prove to be a beneficial strategy for increasing protein synthesis and reducing the likelihood of excess protein “wasting” [7, 8]. This notion is especially important to the aging population as rates of muscle protein synthesis are lower and less responsive to dietary protein ingestion as compared to younger adults [9, 10, and 11].

Since the speculation that pattern of protein intake influences muscle protein synthesis, several studies attempting to determine the optimal quantity of protein required at each meal to maximally stimulate muscle protein synthesis have produced conflicting results. Some randomized trials showed that the consumption of a moderate amount of protein at each meal could stimulate muscle protein synthesis [12], and preserve lean body mass [13] more effectively than consuming most protein at one meal among older adults. Others reported total daily protein intake and pulse feeding (ingesting 80% of daily intake in one meal) to be more beneficial than evenly distributing dietary protein intake throughout the day’s meals [14, 15, and 16]. Of the handful of observational studies examining association between muscle strength, muscle mass or frailty and even distribution of dietary protein across day’s meals [1, 17, 18, and 19], only one was conducted in the United States [19]. This study used data from National Health and Nutrition Examination Survey (NHANES) 1999–2002 and showed positive association between evenly distributed dietary protein and knee extensor strength among U.S. adults aged 50 to 85 year olds [19].

While knee extensor strength measurement requires participant practice and large equipment, isometric grip strength provides a simple, common, and reliable measure of muscle strength [20]. The NHANES provides the nationally representative estimates on isometric grip strength using a handgrip dynamometer. Therefore, we used most recent nationally representative data on grip strength and dietary protein intake to examine the association between intake of at least 25g and 30g protein per eating occasion and total daily protein intake with hand grip strength in U.S. adults aged 51 years and over.

## Methods

### Sample

This study included grip strength and dietary intake data from adults aged 51 years and older in What We Eat in America (WWEIA) NHANES 2011–2012 and 2013–2014. A total of 4,123 individuals (2,025 men and 2,098 women) provided usable grip strength data and complete and reliable dietary intake estimates for one day of dietary intake data. NHANES is a nationally representative, cross-sectional survey on the nutrition and health status of the civilian, non-institutionalized U.S. population conducted by the National Center for Health Statistics (NCHS). Participants complete in-home interviews, physical examinations, dietary interviews, and post examination components. Detailed descriptions of the survey design and data collection procedures are available elsewhere [21, 22]. Oversampling, including that of persons aged 60 years and over, was done to improve the reliability and precision of related estimates [21]. The survey protocol was reviewed and approved by the NCHS Research Ethics Review Board.

### Dietary intake

Dietary intake data were collected by interviewer administered 24-hour recalls using the U.S. Department of Agriculture (USDA) Automated Multiple-Pass Method (AMPM). This method uses a 5-step procedure to quantify 24-hour food and beverage intake. A detailed description of the dietary interview method has been described elsewhere [23, 24]. Protein intake per day and per eating occasion (breakfast, lunch, dinner, and snack) were determined. During the 24-hour dietary recall, a list of eating occasions and their Spanish equivalents was available to the respondent for selection. For each food and beverage reported, respondents were asked to identify the name of the eating occasion. Respondents reporting at least one eating occasion from (1) breakfast; (2) lunch/brunch; (3) dinner/supper; and (4) snack were included in this study. Snack included eating occasions designated by the respondent as snack, drink/beverage, extended consumption or the Spanish equivalent. The frequency of consuming ≥25g and ≥30g of protein across the day’s eating occasions was determined by adding the number of eating occasions during which individuals reported consuming ≥25g and ≥30g of protein. The value for frequency of protein consumption variable ranged from 1–4 (breakfast, lunch/brunch, dinner/supper, and snack). The USDA Food and Nutrient Database for Dietary Studies 2011–2012 and 2013–2014 were used to convert food and beverages consumed into gram amounts and to determine nutrient values [25, 26].

### Hand grip strength

Hand grip strength was measured by hand grip dynamometer, and expressed in kilograms. Participants were excluded from this component if they were unable to hold the dynamometer or only performed the test on one hand. Each hand was tested three times, alternating hands between trials with a 60-second rest between measurements on the same hand. Those who had surgery on either hand/wrist in the last three months were not tested on that particular hand. In our study, we used combined grip strength, calculated as the sum of the largest reading from each hand [27, 28].

### Other variables

Other variables included in this study were age, sex, race/ethnicity, energy intake, physical activity, smoking status, body mass index (BMI) and health status. Race-ethnicity was identified as non-Hispanic white, non-Hispanic black, Hispanic and non-Hispanic Asian. Estimates from day 1 of dietary intake data was used to calculate energy intake (kcal/day). A physical activity questionnaire provided minutes per week spent on exercise. Physical activity was categorized as <150 minutes/week, 150–299 minutes/week, and ≥300 minutes/week [29, 30]. Smoking status was determined as never, former, and current smoker. BMI was calculated based on measured weight and height as weight in kilogram divided by height in meters squared and was categorized as <18.5–24.9 kg/m^2^, 25.0–29.9 kg/m^2^, and ≥30.0 kg/m^2^. The forearm circumference was measured on the right arm at the level of the upper arm mid-point using a measuring tape in centimeters. Self-reported general health status was categorized as excellent/very good, good, or fair/poor.

### Statistical analysis

Population-based estimates for anthropometric, demographic characteristics, and energy intake were generated for participants by quartiles of daily protein intake. The quartile classification was accomplished separately for men and women using sample weights. The proportion of the adults consuming ≥25g protein at various eating occasions was estimated. Grip strength of individuals consuming ≥25g and ≥30g protein at 1 eating occasion was compared to those consuming the same level of protein at 2 and 3 or more eating occasions. For the association between grip strength and daily protein intake, protein intake was examined as total grams intake per day and as grams per kilogram of ideal body weight. Ideal body weight was computed for overweight/obese individuals using Hamwi equation [31]. Grip strength of individuals in quartile 1 of mean daily protein intake was compared to those in each of the other quartiles using survey regression procedures. Associations were examined without and with adjustment for age, sex, race/ethnicity, physical activity, BMI, forearm circumference, health status, and smoking status. BMI and forearm circumference did not affect the association of grip strength with protein intake at various eating occasions and protein intake quartiles, so they were not included in the final model. The comparison involving eating occasions was further adjusted for daily protein intake. The comparison between daily protein intake quartiles was further adjusted for energy intake. Linear orthogonal polynomial contrasts were used to identify the trends in the association between protein intake and grip strength.

Analyses were performed using SAS software, version 9.3 (2010; SAS Institute) and SAS-callable SUDAAN, release 11.0 (2012; RTI International), adjusting for survey design effects resulting from NHANES’ complex, multistage probability sampling [22]. Application of dietary sample weights yielded estimates representative of the U.S. adult population. Significant differences noted within the text are defined by p<0.05.

## Results

The characteristics of the study population by gender-specific quartile of daily protein intake are shown in Tables 1 and 2. The younger men and women (ages 51–60 years old) tended to consume more protein than the older adults (ages 61 years and over). Fewer women in the lowest protein quartile reported excellent/very good health status. Higher protein intake was associated with a higher energy intake and with a greater grip strength in both sexes. Mean daily protein intake was 46.8(0.7)g/d and 141.3(2.1)g/d among men in Q1 and Q4 respectively, and 33.6(0.5)g/d and 104.1(1.4)g/d among women in Q1 and Q4 respectively. Race/ethnicity, smoking status, physical activity, and weight status were not different across protein quartiles by sex.

**Table 1:** Distribution of selected characteristics by quartile of daily protein intake in men ^a^.

	Q1	Q2	Q3	Q4	P value
Men n (%)^d^	619 (30)	503 (25)	459 (23)	444 (22)	
	Mean (SE)				
**Protein intake (g)**	46.8 (0.7)	73.9 (0.4)	96.9 (0.4)	141.3 (2.1)	<0.0001^b^
**Daily energy (kcal/day)**	1541 (33)	2023 (41)	2420 (49)	3235 (50)	<0.0001^b^
**Hand grip strength (kg)**	78.4 (1.5)	81.7 (1.4)	81.9 (0.9)	84.7 (0.9)	0.003 ^b^
	% (SE)				
**Age group**					
51–60 years	39 (3.3)	45 (3.8)	44 (4.0)	56 (2.3)	0.004 ^c^
61 years and older	61 (3.3)	45 (3.8)	56 (4.0)	44 (2.3)	
**Race/ethnicity**					
Non-Hispanic white	71 (3.8)	79 (3.1)	82 (2.4)	77 (2.9)	0.112 ^c^
Non-Hispanic black	15 (2.7)	9 (1.7)	7 (1.3)	10 (1.9)	
Hispanic	9 (1.5)	8 (1.8)	8 (1.4)	9 (2.0)	
Non-Hispanic Asian	5 (0.9)	4 (1.0)	3 (0.9)	3 (0.6)	
**Smoking status**					
Never smoker	39 (2.4)	40 (3.4)	40 (3.9)	43 (3.6)	0.918 ^c^
Former smoker	44 (3.5)	42 (2.7)	45 (3.7)	41 (3.5)	
Current smoker	17 (2.1)	18 (2.8)	15 (1.5)	16 (1.5)	
**Physical activity (minutes/wk)**					
<150	70 (2.5)	70 (3.5)	66 (3.5)	61 (2.4)	0.174 ^c^
150–299	12 (1.7)	11 (2.1)	14 (2.2)	9 (1.7)	
≥300	18 (2.4)	19 (2.8)	20 (3.0)	29 (2.9)	
**Self-reported health status**					
Excellent, very good	37 (2.8)	37 (3.6)	43 (4.3)	39 (3.4)	0.761 ^c^
Good	40 (2.6)	43 (3.7)	39 (3.9)	39 (3.7)	
Fair, poor	23 (1.9)	20 (2.9)	18 (2.6)	23 (3.1)	
**Body mass index (BMI)**					
<18.5–24.9 kg/m^2^	23 (2.9)	25 (3.0)	26 (2.7)	21 (2.9)	0.841 ^c^
25.0–29.9 kg/m^2^	41 (2.9)	36 (2.8)	39 (3.6)	38 (3.6)	
≥30.0 kg/m^2^	36 (2.3)	39 (3.4)	35 (3.3)	40 (3.7)	

^a^ Source: What We Eat in America, National Health and Nutrition Examination Survey 2011–2014, Day 1 dietary intake data, individuals aged 51 years and older.

^b^ P value for comparison across quartiles using ANOVA.

^c^ P value for comparison of the characteristics using chi-square.

^d^ Values are presented as n (%).

**Table 2:** Distribution of selected characteristics by daily protein intake quartile in women ^a^.

	Q1	Q2	Q3	Q4	P value
Women n (%)^d^	602 (29)	518 (25)	488 (23)	490 (23)	
	Mean (SE)				
**Protein intake (g)**	33.6 (0.5)	54.4 (0.3)	71.4 (0.4)	104.1 (1.4)	<0.0001^b^
**Daily energy (kcal/day)**	1124 (18)	1537 (31)	1869 (33)	2308 (41)	<0.0001^b^
**Hand grip strength (kg)**	48.5 (0.7)	50.1 (0.7)	51.2 (0.6)	52.8 (0.5)	0.0006 ^b^
	% (SE)				
**Age**					
51–60 years	37 (2.6)	37 (3.8)	43 (4.5)	52 (2.8)	0.004 ^c^
61 years and older	63 (2.6)	63 (3.8)	57 (4.5)	48 (2.8)	
**Race/ethnicity**					
Non-Hispanic white	73 (3.0)	77 (2.6)	79 (2.5)	77 (3.0)	0.114 ^c^
Non-Hispanic black	15 (2.2)	11 (2.1)	9 (1.4)	9 (1.6)	
Hispanic	8 (1.7)	7 (1.2)	8 (1.6)	9 (1.9)	
Non-Hispanic Asian	4 (0.7)	5 (0.9)	5 (1.0)	4 (0.9)	
**Smoking status**					
Never smoker	56 (3.2)	59 (3.3)	58 (3.2)	54 (3.4)	0.450 ^c^
Former smoker	26 (3.0)	30 (3.0)	25 (3.2)	32 (2.7)	
Current smoker	18 (2.6)	11 (1.6)	16 (4.2)	14 (2.5)	
**Physical activity (minutes/wk)**					
<150	72 (3.1)	70 (3.0)	68 (3.5)	71 (3.0)	0.809 ^c^
150–299	14 (2.5)	14 (2.2)	13 (2.0)	13 (2.2)	
≥300	14 (1.6)	16 (2.3)	19 (3.0)	16 (2.3)	
**Self-reported health status**					
Excellent, very good	33 (2.7)	45 (3.2)	45 (4.0)	45 (3.5)	0.022 ^c^
Good	40 (2.7)	37 (2.3)	32 (2.7)	38 (3.0)	
Fair, poor	27 (2.6)	18 (2.6)	23 (3.2)	17 (2.4)	
**Body mass index (BMI)**					
<18.5–24.9 kg/m^2^	25 (2.5)	30 (2.7)	35 (4.4)	29 (2.3)	0.262 ^c^
25.0–29.9 kg/m^2^	37 (2.6)	26 (2.9)	28 (3.4)	30 (3.3)	
≥30.0 kg/m^2^	38 (2.7)	44 (3.1)	37 (4.5)	40 (3.0)	

^a^ Source: What We Eat in America, National Health and Nutrition Examination Survey 2011–2014, Day 1 dietary intake data, individuals aged 51 years and older.

^b^ P value for comparison across quartiles using ANOVA.

^c^ P value for comparison of the characteristics using chi-square.

^d^ Values are presented as n (%).

About 33% of men and 19% of women had protein intake of ≥25g at two or more eating occasions, whereas 13% of men and 31% of women reported not consuming this amount at any of the eating occasions. Also, Grip strength, daily protein intake, and protein intake at breakfast, lunch, dinner and snacks were significantly higher among men and women reporting consumption of ≥25g protein at two or more eating occasions as compared to those consuming this amount at 1 eating occasion (Table 3).

**Table 3:** Grip strength, daily protein intake, and protein intake at breakfast, lunch, dinner, and snacks by intake of ≥25g protein at various eating occasions ^a^.

	Intake of ≥25 g protein at			
	0 eating occasion	1 eating occasion (reference group)	2 eating occasions	≥3 eating occasions
Men n (%)^c^	271 (13.4)	936 (46.2)	674 (33.3)	144 (7.1)
Hand grip strength (kg) ^d^	77.6 (1.8)	80.6 (1.2)	83.9 (0.7)^b^	83.3 (1.9)
Daily protein intake (g/day)^d^	40.6 (1.5)^b^	76.1 (1.5)	108.3 (1.7)^b^	145.7(4.6)^b^
Breakfast (g/day)^d^	8.4 (0.6)^b^	12.0 (0.4)	18.3 (0.8)^b^	29.9 (2.2)^b^
Lunch/ brunch (g/day)^d^	10.2 (1.1)^b^	18.6 (1.2)	30.6 (1.3)^b^	38.5 (2.3)^b^
Dinner/ supper (g/day)^d^	15.3 (0.6)^b^	36.4 (1.6)	46.5 (1.3)^b^	51.8 (3.9)^b^
Snacks (g/day)^d^	6.6 (0.6)^b^	9.1 (0.7)	12.8 (0.8)^b^	25.5 (3.3)^b^
Women n (%)^c^	652 (31.1)	1012 (48.2)	391 (18.6)	43 (2.0)
Hand grip strength (kg)^d^	49.5 (0.7)	50.4 (0.5)	53.0 (0.6)^b^	49.3 (2.8)
Daily protein intake (g/day)^d^	39.9 (0.9)^b^	66.1 (0.9)	97.0 (1.8)^b^	128.7 (4.7)^b^
Breakfast (g/day)^d^	8.9 (0.4)^b^	11.0 (0.4)	14.0 (0.8)^b^	24.4 (2.6)^b^
Lunch/ brunch (g/day)^d^	10.3 (0.4)^b^	17.1 (0.7)	31.8 (1.0)^b^	36.2 (2.9)^b^
Dinner/ supper (g/day)^d^	14.6 (0.4)^b^	30.7 (0.8)	40.0 (1.4)^b^	44.5 (4.1)^b^
Snacks (g/day)^d^	6.2 (0.4)	7.3 (0.4)	11.2 (0.9)^b^	23.7 (4.3)^b^

^a^ Source: What We Eat in America, National Health and Nutrition Examination Survey 2011–2014, Day 1 dietary intake data, individuals aged 51 years and older.

^b^ Significant difference (p<0.05) from reference group (≥25g protein at 1 eating occasion).

^c^ Values are presented as n (%).

^d^ Values are provided as mean (SE).

As compared to consumption of ≥25g or ≥30g protein at 1 eating occasion, grip strength was positively associated with consumption of same level of protein at more than 1 eating occasions in unadjusted model (model 1) but not after adjustment for confounders (model 2) in both men and women(Table 4).

**Table 4:** Association between grip strength and intake of ≥25g and ≥30g protein at 1 vs. 2, and 3 or more eating occasions before and after adjusting for potential confounders ^a^.

	**Intake of ≥25g protein at**		
	**1 vs 2 eating occasions**	**1 vs ≥3 eating occasions**	**2 vs ≥3 eating occasions**
Men (n = 2025)			
Model 1 (Unadjusted)	3.3 (1.2) ^b^	2.7 (1.8)	-0.6 (1.7)
Model 2 (Adjusted)	0.8 (1.2)	-0.3 (1.7)	-1.1 (1.6)
Women (n = 2098)			
Model 1 (Unadjusted)	2.6 (0.9) ^b^	-1.0 (2.7)	-3.6 (3.0)
Model 2 (Adjusted)	1.1 (0.8)	-3.2 (1.8)	-4.3 (2.2)
	**Intake of ≥30g protein at**		
	**1 vs 2 eating occasions**	**1 vs ≥3 eating occasions**	**2 vs ≥3 eating occasions**
Men (n = 2025)			
Model 1 (Unadjusted)	3.6 (1.4)	5.1 (2.1) ^b^	1.5 (2.0)
Model 2 (Adjusted)	1.3 (1.3)	2.6 (1.8)	1.3 (1.9)
Women (n = 2098)			
Model 1 (Unadjusted)	2.7 (1.1) ^b^	-1.7 (3.8)	-4.4 (3.8)
Model 2 (Adjusted)	1.5 (1.1)	-2.9 (2.3)	-4.3 (2.4)

^a^ Source: What We Eat in America, National Health and Nutrition Examination Survey 2011–2014, Day 1 dietary intake data, individuals aged 51 years and older.

^b^ P<0.05

Model 2 adjusted for age, race/ethnicity, smoking status, self-reported health status, energy adjusted protein intake (residual method), and physical activity. BMI and forearm circumference did not change the associations and were not included in the final analyses.

All values in models 1 and 2 are mean difference (SE).

Grip strength was positively associated with mean daily protein intake in men and women with protein intake in quartiles 3 and 4 as compared to those with protein intake in quartile 1 before adjustment for confounders (model 1). The association remained significant after adjustment for confounders only in women (model 2). There was a significant linear trend in the association between grip strength and daily protein intake in both men and women before adjustment for potential confounders (model 1), but not after adjustment (model 2) (Table 5). There was no association between grip strength and protein intake quartiles, when daily protein intake was considered as gram per kilogram of ideal body weight.

**Table 5:** Association between grip strength and daily protein intake before and after adjusting for potential confounders ^a^.

	Quartiles of protein intake				
	Q1	Q2	Q3	Q4	P for trend
Men (n = 2025)	619	503	459	444	
Grip strength (kg)^c^	78.4 (1.5)	81.7 (1.4)	81.9 (0.9)	84.7 (0.9)	
Model 1 (Unadjusted)	1	3.2 (1.8)	3.5 (1.4) ^b^	6.3 (1.4) ^b^	<0.0001
Model 2 (Adjusted)	1	2.0 (1.6)	1.3 (1.6)	2.8 (1.6)	0.115
Women (n = 2098)	602	518	488	490	
Grip strength (kg) ^c^	48.5 (0.7)	50.1 (0.7)	51.2 (0.6)	52.8 (0.5)	
Model 1 (Unadjusted)	1	1.6 (1.0)	2.7 (0.9) ^b^	4.3 (0.9) ^b^	0.006
Model 2 (Adjusted)	1	1.7 (0.9)	2.1 (0.8) ^b^	2.4 (0.9) ^b^	0.195

^a^ Source: What We Eat in America, National Health and Nutrition Examination Survey 2011–2014, Day 1 dietary intake data, individuals aged 51 years and older.

^b^ P<0.05

^c^ Values are mean (SE)

Model 2 adjusted for age, race/ethnicity, smoking status, self-reported health status, energy intake, and physical activity. BMI and forearm circumference did not change the associations and were not included in the final analyses.

All values in models 1 and 2 are mean difference (SE).

## Discussion

This study examined the association between grip strength and protein intake of ≥25g and ≥30g per eating occasion and total daily protein intake using a nationally representative sample of U.S. adults. Our results show that more frequent consumption of meals containing ≥25g and ≥30g was not associated with grip strength after adjusting for potential confounders. However, higher daily protein intake was positively associated with grip strength in women but not in men after adjustment for potential confounders.

Previous randomized trials on the effect of evenly distributing protein intake and muscle strength among older adults produced conflicting results. The quantity, but not the protein intake pattern (even or uneven across breakfast, lunch, and dinner) contributed to greater muscle protein synthesis in a study among older adults aged 52–75 years [14]. Whereas, protein pulse feeding improved protein retention as compared to a protein-spread pattern in healthy and hospitalized older adults aged 68 and 85 years, respectively [15, 16]. A few observational studies reported positive association between even distribution of protein across day’s meals and higher composite muscle strength score [1, 17, and 18]. It is difficult to compare our findings with these studies because of differences in study design (longitudinal versus cross-sectional in our study) [17, 18], outcome (frailty and composite muscle strength score versus grip strength in our study) [1, 18] and age of the study population (75 years and over versus 51 years and over in our study). Recently, a study based on NHANES data from 1999–2002 on adults reported positive association between consumption of higher frequency of meals containing at least 30g protein and knee extension strength [19]. We did not find similar association between intake of at least 25 and 30g protein and grip strength. The disparity between the previous NHANES study and our study results may be attributed to differences in outcome variable (knee extensor strength vs. grip strength in our study). It is also possible that the lack of association between consumption of at least 25-30g protein at 3 or more eating occasions and grip strength in our study may be due to small sample size for this group (n = 144(7%) in men and 43(2%) in women).

A few cross-sectional studies have examined associations between daily protein intake and handgrip strength. Gregorio et. al., found no difference in mean grip strength between 73 years old women consuming <0.8 g protein per kg body weight and those consuming more. However in that study, there was a significant difference when the outcome variable was a composite physical performance test score [5]. In a study on community dwelling older adults aged 75 years and older, there was no difference in the risk of low handgrip strength in quartiles of higher protein intake compared to the quartile with the lowest intake [1]. The difference between our findings and those of previous cross-sectional studies may be attributed to our data stratified by gender while the other studies pooled their analysis for men and women. The exact mechanism behind the gender-wise disparity in the association between grip strength and protein intake quartiles in our study is not clear. As compared to women, a rapid age-related decline in physical function and grip strength was observed in men despite their higher initial levels of muscle strength score and grip strength in a Danish cohort study [32]. The gender difference in the association between grip strength and protein intake observed in our study may be explained by sex differences in grip strength, muscle mass and hormonal factors [32, 33, and 34]. More studies are needed to determine the exact mechanism explaining effect of gender disparity on the association between protein intakes and grip strength.

In our study, the average protein intake in the highest quartile was 141g and 104g in men and women, which is more than twice the recommended amount of protein per day for adult men and women, respectively. Adults aged 52–75 years consuming twice the recommended amount of protein per day have been shown to have greater positive nitrogen balance in whole body protein kinetics study [14]. It is possible that positive nitrogen balance resulting from higher protein intake may partly account for the association between grip strength and higher protein intake. The results of our study are consistent with those of longitudinal studies that show a positive relationship between daily protein intake and grip strength [17, 18, 35, and 36].

Our study has some methodological limitations. NHANES data are restricted to the non-institutionalized population, the results from this study cannot be generalized to institutionalized populations. Use of self-reported dietary intakes may introduce bias due to misreporting. However, the AMPM has undergone extensive testing and has been validated providing reliable estimates for total energy intake at the group level [23]. Muscle mass and hand length data were unavailable in NHANES 2012–2014 and therefore, we were unable to determine if the association between grip strength and total protein intake in women could be explained by muscle mass or hand length. The major strengths of this study include use of a relatively large nationally representative sample of U.S. adults aged 51 years and over and detailed data on dietary intake and eating occasions which allowed us to examine distribution of protein intake over the day.

## Conclusion

The rapidly rising population of older adults and potential increase in age associated physical disability warrants further research to understand the role of dietary protein in muscle preservation and strength. In a nationally representative sample of older adults, consuming ≥25g or ≥30g protein at 2 or more eating occasions was not associated with hand grip strength. However, higher daily protein intake was positively associated with higher hand grip strength in women but not in men. Future studies are needed to confirm the gender disparity and its implications.
